# *Haemophilus influenzae* Infection Drives IL-17-Mediated Neutrophilic Allergic Airways Disease

**DOI:** 10.1371/journal.ppat.1002244

**Published:** 2011-10-06

**Authors:** Ama-Tawiah Essilfie, Jodie L. Simpson, Jay C. Horvat, Julie A. Preston, Margaret L. Dunkley, Paul S. Foster, Peter G. Gibson, Philip M. Hansbro

**Affiliations:** 1 Centre for Asthma and Respiratory Diseases and Hunter Medical Research Institute, The University of Newcastle, Newcastle, New South Wales, Australia; 2 Department of Respiratory and Sleep Medicine, John Hunter Hospital, New Lambton, New South Wales, Australia; 3 Hunter Immunology, Newcastle, Australia; University of Toronto, Canada

## Abstract

A subset of patients with stable asthma has prominent neutrophilic and reduced eosinophilic inflammation, which is associated with attenuated airways hyper-responsiveness (AHR). *Haemophilus influenzae* has been isolated from the airways of neutrophilic asthmatics; however, the nature of the association between infection and the development of neutrophilic asthma is not understood. Our aim was to investigate the effects of *H. influenzae* respiratory infection on the development of hallmark features of asthma in a mouse model of allergic airways disease (AAD). BALB/c mice were intraperitoneally sensitized to ovalbumin (OVA) and intranasally challenged with OVA 12–15 days later to induce AAD. Mice were infected with non-typeable *H. influenzae* during or 10 days after sensitization, and the effects of infection on the development of key features of AAD were assessed on day 16. T-helper 17 cells were enumerated by fluorescent-activated cell sorting and depleted with anti-IL-17 neutralizing antibody. We show that infection in AAD significantly reduced eosinophilic inflammation, OVA-induced IL-5, IL-13 and IFN-γ responses and AHR; however, infection increased airway neutrophil influx in response to OVA challenge. Augmented neutrophilic inflammation correlated with increased IL-17 responses and IL-17 expressing macrophages and neutrophils (early, innate) and T lymphocytes (late, adaptive) in the lung. Significantly, depletion of IL-17 completely abrogated infection-induced neutrophilic inflammation during AAD. In conclusion, *H. influenzae* infection synergizes with AAD to induce Th17 immune responses that drive the development of neutrophilic and suppress eosinophilic inflammation during AAD. This results in a phenotype that is similar to neutrophilic asthma. Infection-induced neutrophilic inflammation in AAD is mediated by IL-17 responses.

## Author Summary

Approximately 50% of asthmatics have non-eosinophilic inflammation, and 20% of these patients have severe neutrophilic inflammation and increased IL-8 levels. These so-called neutrophilic asthmatics have persistent airway colonization with bacteria, and *Haemophilus influenzae* is one of the bacteria most commonly isolated. However, how *H. influenzae* is associated with the pathogenesis of neutrophilic asthma is unknown. In this study we used mouse models to investigate the relationship between *H. influenzae* infection and allergic airways disease (AAD). We showed that infection promoted the development of hallmark features of neutrophilic asthma. Infection suppressed Th2 cytokines, eosinophilic inflammation, and AHR in AAD, while increasing neutrophilic inflammation and IL-17 responses. Importantly, inhibition of IL-17 during AAD reduced airway neutrophils and neutrophil chemokines, suggesting that infection drives the development of neutrophilic inflammation through an IL-17-mediated mechanism. This provides novel insights into the mechanisms that may underpin infection-induced neutrophilic asthma. These data also suggest that treatments targeting infection may lead to improved management of neutrophilic asthma.

## Introduction

Asthma is a complex disease of the airways that is generally characterized by symptoms of wheeze, cough, breathlessness and airway inflammation. While eosinophilic inflammation has been considered to be the hallmark of airway inflammation in asthma [1], [2], it is present in only 50% of asthmatics [3]. Non-eosinophilic asthma has now been described in persistent [4], [5] and severe asthma, [6] as well as in steroid naïve asthma [7]. Further investigation of the non-eosinophilic subtype has identified a subgroup with an intense neutrophilic bronchitis [5], [8] with increased interleukin (IL)-8 [4]. Compared to eosinophilic asthmatics, neutrophilic asthmatics have reduced eosinophilic inflammation and AHR. Furthermore, they are frequently resistant to corticosteroid treatment, which results in a significant proportion of asthma-related health care costs [5], [8], [9], [10], [11], [12]. IL-17 is also elevated in asthma and other obstructive airway diseases that are characterized by increased neutrophils [13], [14], [15], [16].

IL-8 and IL-17 are important mediators of neutrophilic inflammation during infection and in disease states [4], [12], [13], [17], [18] and their elevated expression in neutrophilic asthma correlates with increased levels of neutrophils in sputum [15]. IL-8 is a potent neutrophil chemoattractant, produced by macrophages, lymphocytes, epithelial cells and neutrophils [19], [20]. IL-17 is produced by several cells including Th17 cells [21], [22], [23], γδ T cells [24], [25], neutrophils [26], and macrophages [27], [28]. IL-17 has critical roles in host defence against bacterial infections [29], [30], [31], [32], suggesting a potential role in the pathogenesis of bacterial-induced neutrophilic asthma.

Chronic bacterial colonization is evident in the airways of patients with neutrophilic asthma [12] and is also associated with an intense neutrophilic bronchitis in asthma [33]. *H. influenzae* is a common bacterium of the respiratory tract, is one of the bacteria most frequently isolated from the airways of neutrophilic asthmatics [12], [33], and often causes recurrent respiratory disease [34], [35], [36] in those with compromised airways. Nevertheless, how *H. influenzae* is associated with the pathogenesis of neutrophilic asthma is unknown. Specifically, whether infection promotes the pathogenesis of neutrophilic asthma, or if neutrophilic asthmatics are predisposed to infection is not known.

In this study we used murine models of *H. influenzae* infection and OVA-induced allergic airways disease, which mimics hallmark features of human asthma, to elucidate the potential association between infection and the development of neutrophilic asthma.

## Results

### Non-typeable Haemophilus influenzae infection

**Figure 1:**
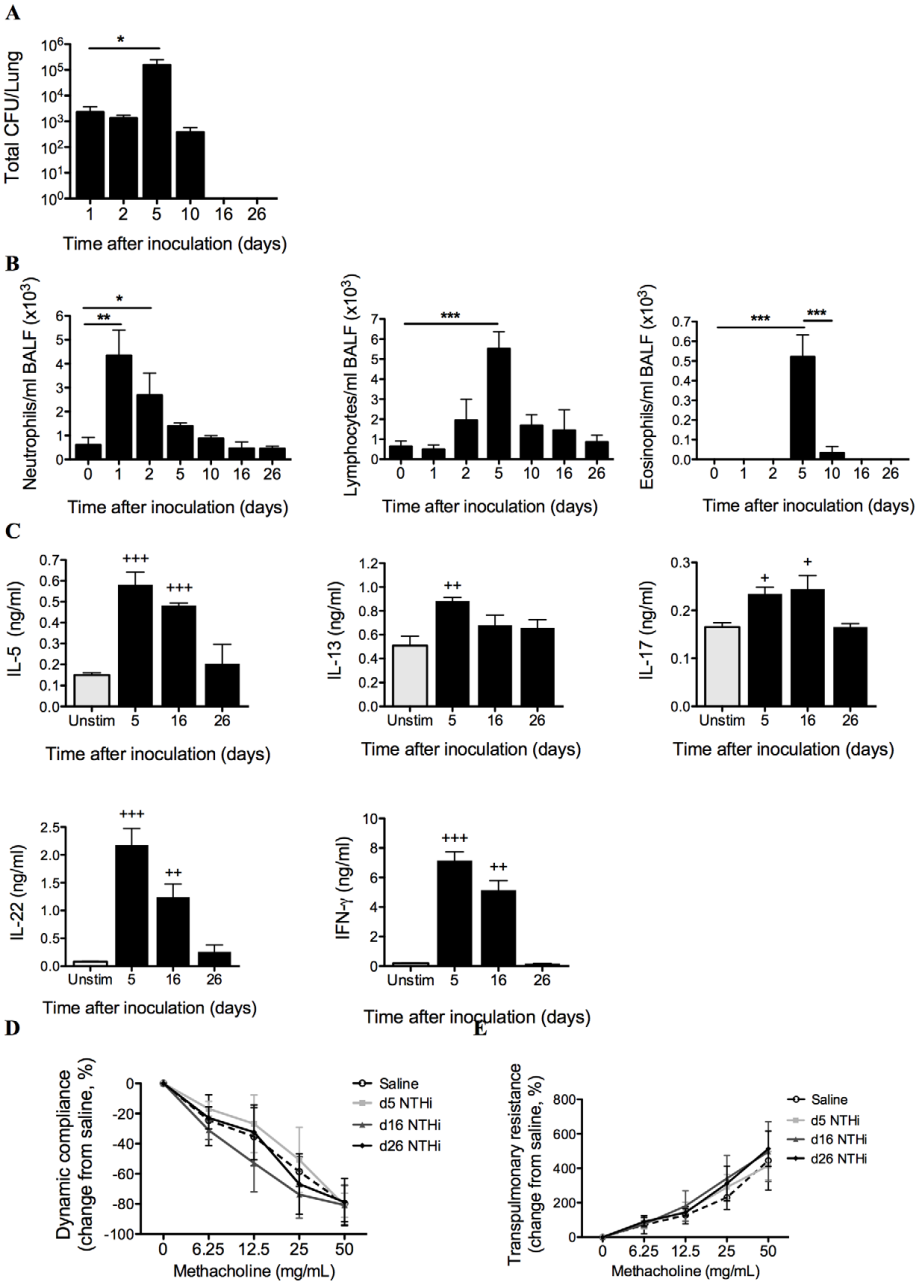
Characterization of NTHi infection. The profile of infection was assessed in mice that only received NTHi (i.e. not OVA), by performing a time-course of bacterial recovery from BALF and lung homogenates (A), and airway inflammation represented by BALF neutrophils, lymphocytes and eosinophils (B). IL-5, IL-13, IL-17, IL-22 and IFN-γ (C) release from MLN T cells stimulated with killed NTHi compared to unstimulated cells was also determined. Lung function in terms of AHR (dynamic compliance (D) and transpulmonary resistance (E)) in response to increasing doses of methacholine was assessed 5, 16 and 26 days after inoculation. N.B. P values for compliance and resistance were determined for the entire dose response curve, *** p<0.001, ** p<0.01, * p<0.05 compared to indicated time points, +++ p<0.001, ++ p<0.01, + p<0.05 compared to unstimulated controls.

In order to investigate the association between *H. influenzae* lung infection and asthma we first established and characterized a murine model of NTHi lung infection alone. Inoculation intratracheally (i.t.) with 5x10^5^ CFU of NTHi resulted in a mild respiratory infection that induced inflammatory responses but did not significantly affect lung function (Figure 1).

Bacterial numbers in bronchoalveolar lavage fluid (BALF) and lung homogenates peaked at 5 days and were cleared 16 days after inoculation (Figure 1A). NTHi infection induced airway inflammation. Neutrophil influx into the airways peaked at 24 hours post-infection while lymphocytes and eosinophils were significantly increased at 5 days. Neutrophil numbers returned to baseline after 5 days, while lymphocyte and eosinophil numbers returned to baseline after 10 days post-infection (Figure 1B). Infection also induced significant but low level increases in NTHi-induced IL-5, IL-13, IL-17 and IL-22, and higher levels of IFN-γ release from mediastinal lymph nodes (MLN) cultures after 5 days, which returned to baseline levels after 26 days (Figure 1C). Infection did not affect lung function, with no changes in AHR (dynamic compliance or transpulmonary resistance in response to increasing doses of methacholine) compared to sham infected (Saline) controls 5, 16 and 26 days after inoculation (Figure 1D–E).

### NTHi infection suppresses key features of Th2-driven eosinophilic AAD

**Figure 2:**
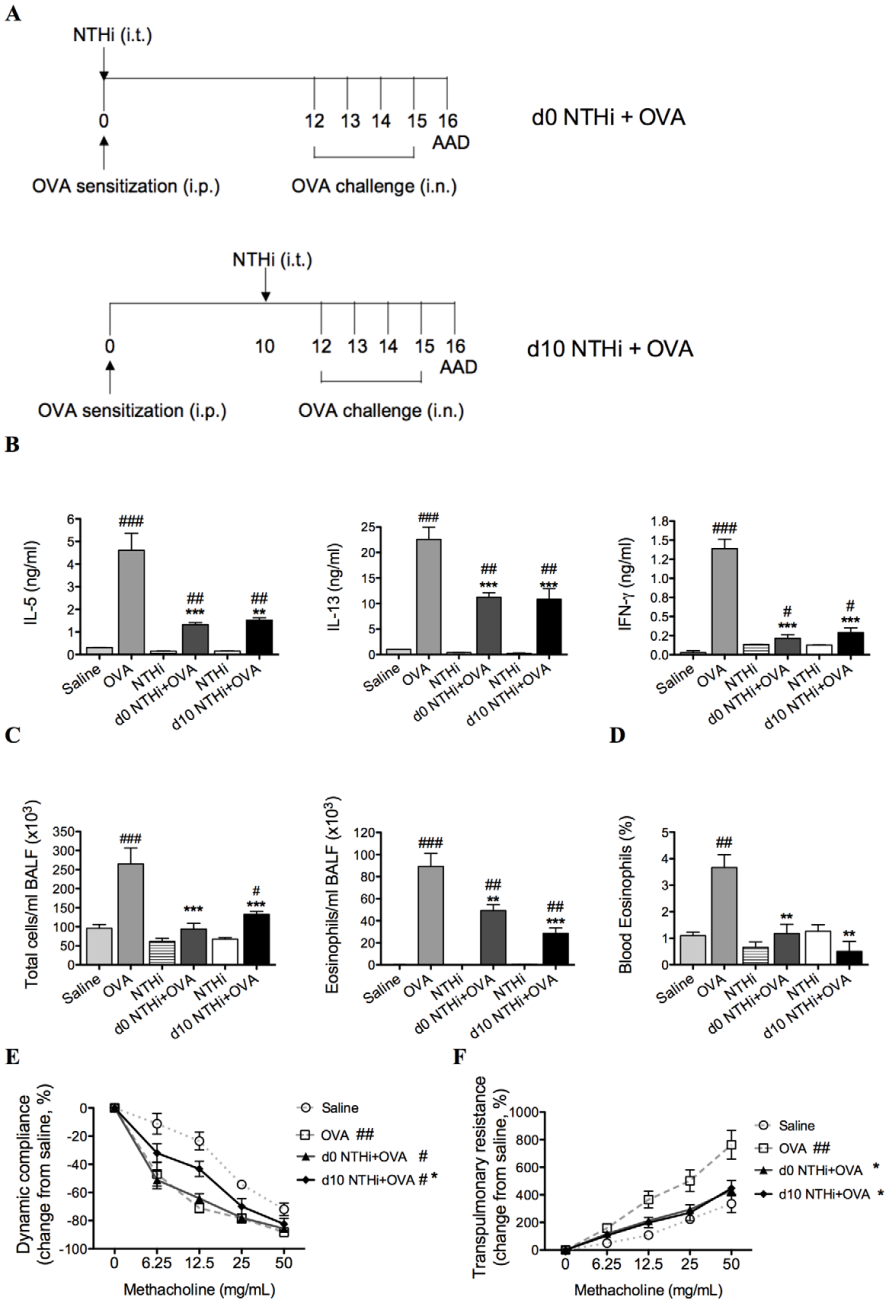
NTHi infection suppressed key features of Th2-driven eosinophilic AAD. Groups of mice were infected during (d0 NTHi+OVA) or 10 days after (d10 NTHi+OVA) OVA sensitization (A), and AAD was analyzed (on day 16). The effects of infection on IL-5, IL-13, and IFN-γ (B) release from MLN T cells stimulated with OVA, BALF total cell and eosinophil counts (C) and the percentage of blood eosinophils (D) were assessed. AHR in terms of dynamic compliance (E) and transpulmonary resistance (F) was determined (see figure 1D-E for infection only AHR results). Data for the corresponding NTHi control groups were obtained at the same time point after infection as data from the NTHi+OVA groups. N.B. P values for compliance and resistance were determined for the entire dose response curve, ### p<0.001, ## p<0.01, # p<0.05 compared to Saline controls, *** p<0.001, ** p<0.01, * p<0.05 compared to OVA controls.

To investigate the effect of infection on AAD in sensitized animals, groups were infected during (d0 NTHi+OVA) or 10 days after (d10 NTHi+OVA) OVA sensitization (Figure 2A), and AAD assessed on day 16. Infection suppressed OVA-induced T cell cytokine responses, inflammatory cell influx and AHR in AAD (Figure 2).

**Figure 3:**
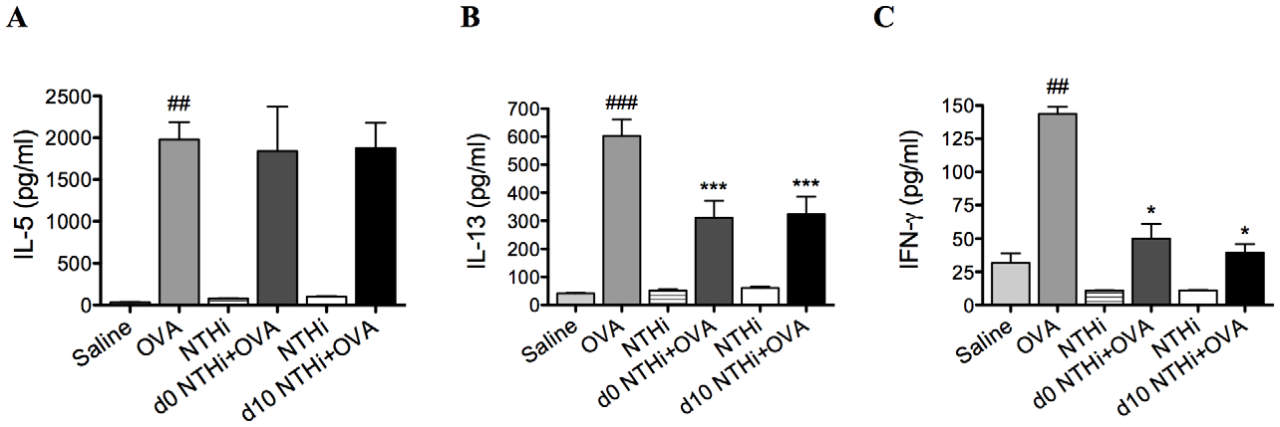
NTHi infection suppressed systemic IL-13 and IFN-γ responses in AAD. Groups of mice were infected during (d0 NTHi+OVA) or 10 days after (d10 NTHi+OVA) OVA sensitization, and AAD was analyzed (on day 16). The effect of infection on IL-5 (A), IL-13 (B), and IFN-γ (C) release from splenocytes stimulated with OVA was assessed. Data for the corresponding NTHi control groups were obtained at the same time point after infection as data from the NTHi+OVA groups. ### p<0.001, ## p<0.01 compared to Saline controls, *** p<0.001, * p<0.05 compared to OVA controls.

The development of AAD (OVA groups) resulted in increased OVA-induced release of IL-5, IL-13 and IFN-γ from MLN and splenic T cells, eosinophilic inflammation and AHR (decreased compliance and increased resistance in response to methacholine), compared to uninfected, nonallergic (Saline) controls (Figure 2B–F and Figure 3). Infection during (d0 NTHi+OVA) or after (d10 NTHi+OVA) sensitization resulted in significant reductions in OVA-induced IL-5, IL-13 and IFN-γ release from MLN T cells (Figure 2B), compared to uninfected, allergic (OVA) controls. Infection also significantly reduced the numbers of total cells and eosinophils in the airways and blood (Figure 2C-D). The reduction in eosinophils correlated with the reduced release of IL-5 from MLN T cells. Infection significantly suppressed, but did not abolish AHR in AAD. Infection during sensitization (d0 NTHi+OVA) had no effect on compliance, but significantly reduced resistance of the lungs. However, infection after sensitization (d10 NTHi+OVA), significantly suppressed both compliance and resistance (Figure 2E–F). Notably, compliance remained decreased in infected, allergic (NTHi+OVA) groups compared to uninfected, nonallergic (Saline) controls.

### NTHi infection suppresses systemic responses in AAD

Infection during (d0 NTHi+OVA) or after (d10 NTHi+OVA) sensitization had no effect on systemic IL-5 (Figure 3A) but significantly reduced systemic IL-13 and IFN-γ release from splenocytes (Figure 3B–C), compared to uninfected, allergic (OVA) controls.

### T regulatory cells (Tregs) are not involved in the suppression of AAD

**Figure 4:**
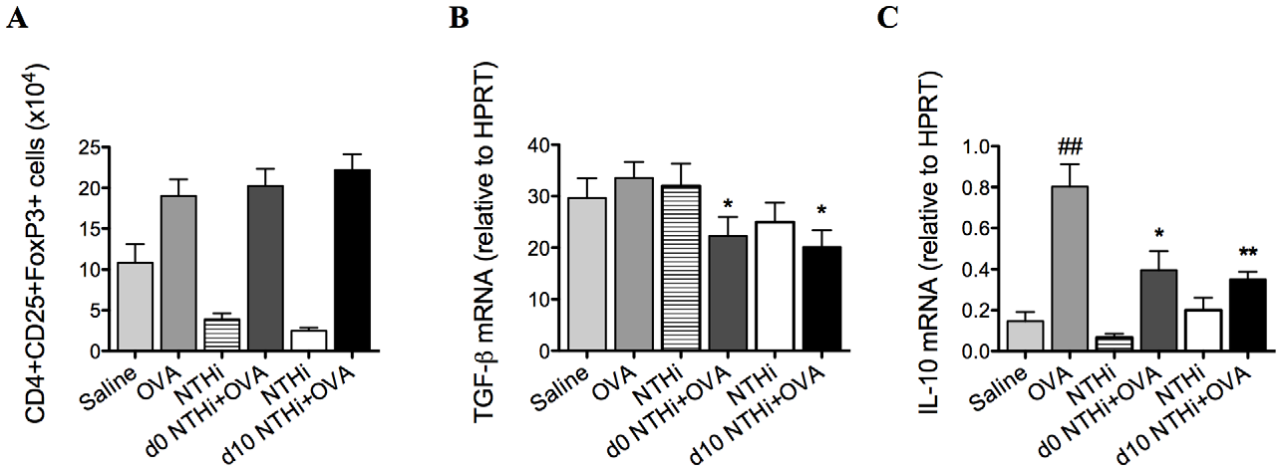
NTHi infection does not affect T regulatory cells in the suppression of AAD. Treg numbers in the lung (A), TGF-β (B) and IL-10 (C) mRNA expression in lung tissue were assessed (on day 16). Data for the corresponding NTHi control groups were obtained at the same time point after infection as data from the NTHi+OVA groups. ## p<0.01, compared to Saline controls, ** p<0.01, * p<0.05 compared to OVA controls.

To determine if Tregs were involved in the suppression of AAD, Tregs, TGF-β and IL-10 were quantified on day 16 of the model. TGF-β and IL-10 are critical immunosuppressive factors that are produced by Tregs. NTHi infection during and after sensitization did not alter the numbers of Tregs (Figure 4A) in the lung, compared to uninfected allergic controls. Notably, infection decreased the expression of TGF-β (Figure 4B) and IL-10 (Figure 4C) in lung tissue in infected, allergic groups compared to uninfected, allergic controls.

### Infection reduced markers of antigen presentation and co-stimulation in the suppression of AAD

**Figure 5:**
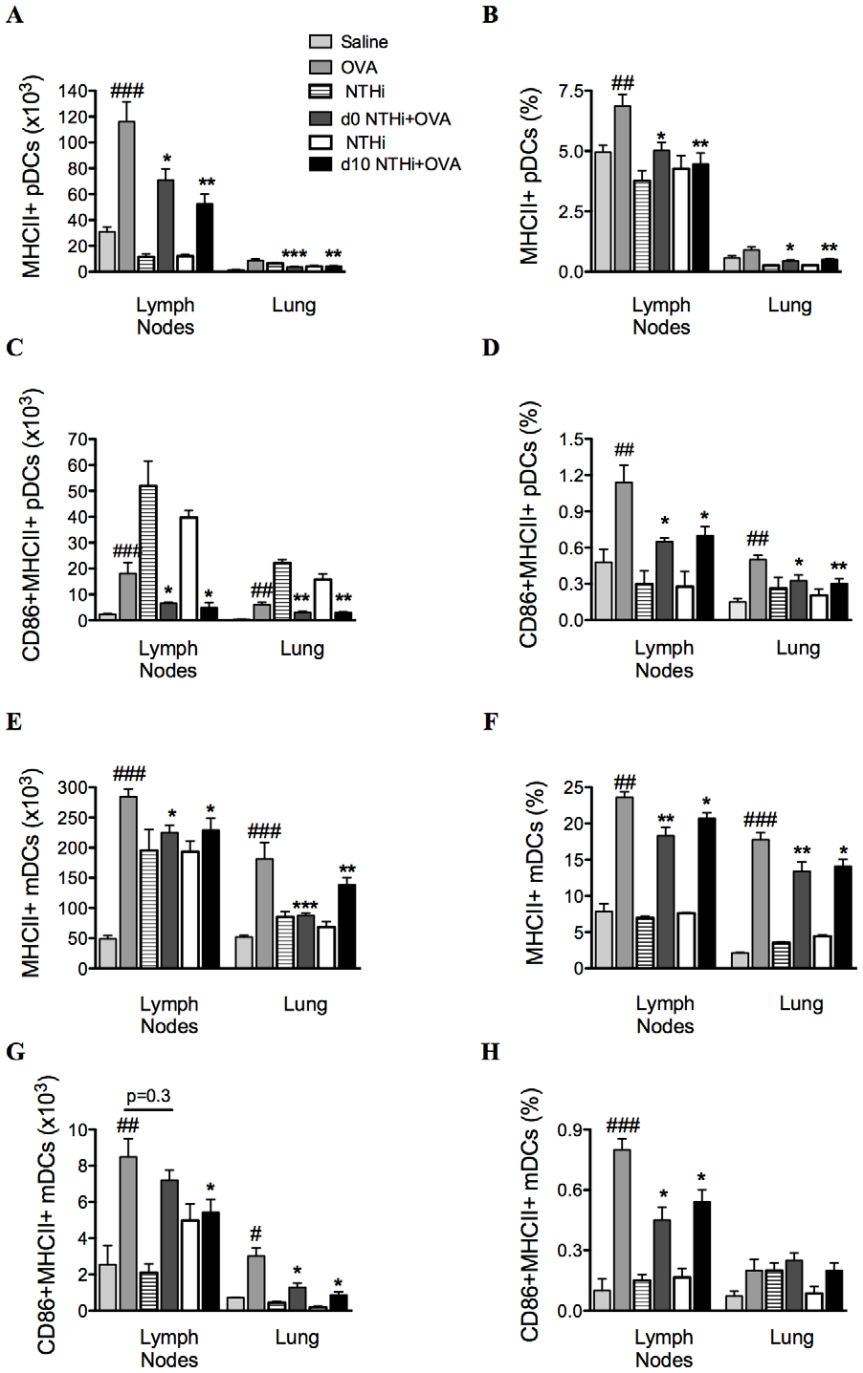
NTHi infection reduced markers of antigen presentation and activation in AAD. The numbers and proportions of MHCII and CD86 expressing pDCs (A–D) and mDCs (E–H) in MLNs and lungs were determined. Data for the corresponding NTHi control groups were obtained at the same time point after infection as data from the NTHi+OVA groups. ###p<0.001, ## p<0.01, # p<0.05 compared to Saline controls, ***p<0.001, ** p<0.01, * p<0.05 compared to OVA controls.

To determine if alterations in antigen-presenting cells were involved in the suppression of AAD, the effect of infection on MHCII and CD86 expressing DCs was also investigated (on day 16). The development of AAD resulted in increases in the numbers and proportions of MHCII expressing plasmacytoid DCs (pDCs) and myeloid DCs (mDCs), and CD86 expressing MHCII+ pDCs and mDCs in MLNs and lungs (Figure 5A–H), compared to uninfected, nonallergic controls. Infection during or after sensitization resulted in significant decreases in MHCII and CD86 expressing pDCs and mDCs compared to uninfected, allergic controls (Figure 5A–H).

### Infection enhances neutrophilic inflammation in AAD

We then assessed the effects of infection on other features of AAD (on day 16). Significantly, whilst eosinophilic inflammatory responses were suppressed by infection during AAD, NTHi infection induced AAD with an enhanced neutrophilic inflammatory profile.

**Figure 6:**
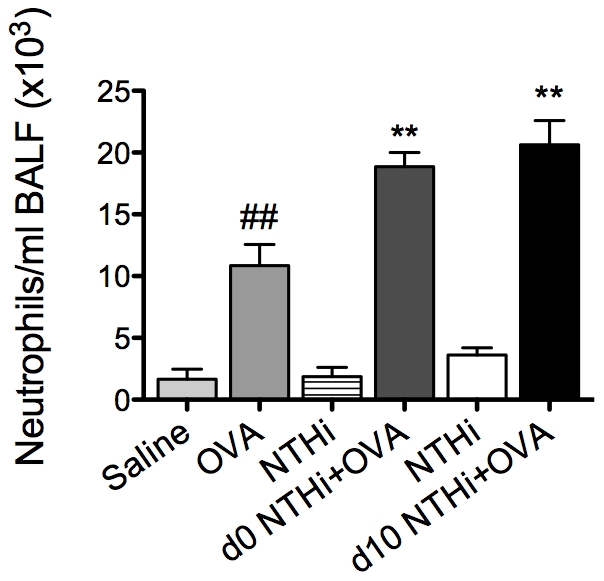
NTHi infection induces neutrophilic inflammation in AAD. The effect of NTHi infection on BALF neutrophil numbers in AAD was determined (on day 16) and compared to groups with AAD without infection or infection alone without AAD. Data for the corresponding NTHi control groups were obtained at the same time point after infection as data from the NTHi+OVA groups. ## p<0.01 compared to Saline controls, ** p<0.01 compared to OVA controls.

The development of AAD resulted in an increase in neutrophil influx into the airways (Figure 6). Infection during or after sensitization resulted in a two-fold increase in neutrophil recruitment in BALF compared to uninfected, allergic controls. Moreover, infected allergic groups had a four-fold increase in neutrophil recruitment compared to groups with infection alone (i.e. infected, nonallergic groups) at the same time point after infection (i.e. 16d and 5d after infection, Figure 6). These results demonstrate that the combination of infection with AAD results in enhanced neutrophilic inflammation. Collectively, our results show that NTHi infection in AAD may induce a phenotype of neutrophilic AAD that resembles neutrophilic asthma in humans.

### Infection-induced neutrophilic inflammation is associated with increases in IL-17 responses

Neutrophilic inflammation in asthma has been linked with increased IL-17 expression and IL-17 has been shown to be involved in neutrophil recruitment in response to bacterial infection. Therefore, the effects of infection on IL-17 responses during infection-induced neutrophilic AAD were further investigated. The experiments described hereafter were performed with infection during (d0 NTHi+OVA) OVA sensitization.

**Figure 7:**
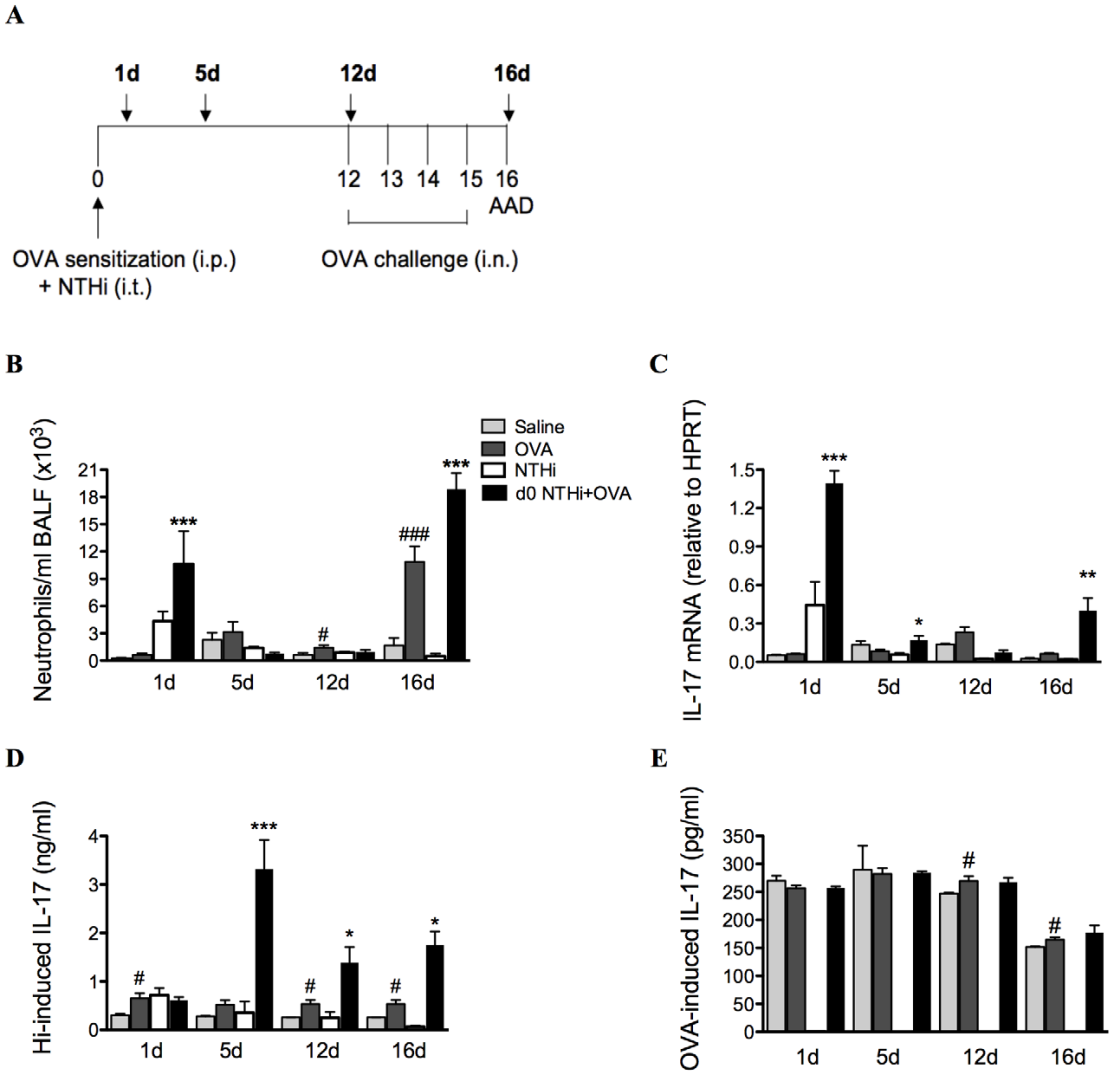
NTHi infection induces increased IL-17 responses that correlate with neutrophil influx in neutrophilic AAD. To investigate the association between neutrophil influx and IL-17 responses, a time-course (A), of neutrophils in BALF (B), IL-17 mRNA expression in lung tissue (C), and IL-17 release from MLN T cells stimulated with killed NTHi (D), and OVA (E) was assessed. Data for the corresponding NTHi control groups were obtained at the same time point after infection as data from the NTHi+OVA groups. ### p<0.001, # p<0.05 compared to Saline controls, *** p<0.001, ** p<0.01, * p<0.05 compared to OVA controls.

The profile of neutrophil influx into the airways and IL-17 production was determined in lung tissue and MLNs during the development of infection-induced neutrophilic AAD (1, 5, 12 and 16d, Figure 7A). The development of AAD resulted in increases in neutrophilic influx into the airways on day 12 and 16, and had minimal effects on IL-17 responses. Significantly greater numbers of neutrophils were recruited into the airways during infection and OVA sensitization (1d) and after OVA challenge (16d) in infected, allergic compared to uninfected, allergic groups (Figure 7B).

Importantly, increases in the infection-induced neutrophil influx were accompanied by significant increases in IL-17 responses in pulmonary tissue and MLN T cells. The expression of IL-17 mRNA in lung tissue was significantly elevated 1 day after infection in infected, allergic groups (Figure 7C) and returned to baseline levels by day 12, immediately prior to OVA challenge. Expression again significantly increased on day 16, after OVA challenges.

NTHi-induced IL-17 release from MLN T cells was also increased from days 5 to 16 in infected, allergic compared to uninfected, allergic groups (Figure 7D). Interestingly, infection did not affect OVA-induced IL-17 release (Figure 7E). Taken together, these data demonstrate that infection induces increased IL-17 responses in lung tissue and MLNs that correlate with elevated airway neutrophil numbers in infection-induced neutrophilic AAD.

### Infection-induced early influx of neutrophils is associated with early increases in neutrophil chemokine responses

**Figure 8:**
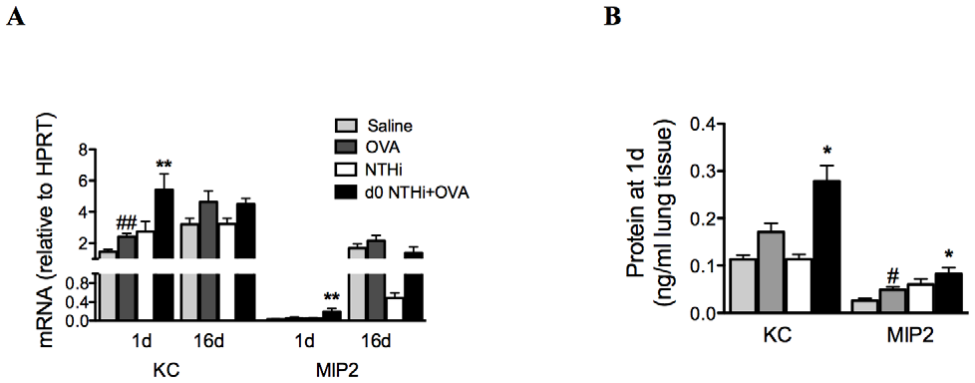
Early neutrophil influx is associated with enhanced neutrophil chemokine expression. To investigate the association between early neutrophil influx and neutrophil chemokine responses, KC and MIP2 mRNA expression (on day 1 and 16, A) and protein levels (day 1 only, B) in lung tissue were assessed. Data for the corresponding NTHi control groups were obtained at the same time point after infection as data from the NTHi+OVA groups. ## p<0.01, # p<0.05 compared to Saline controls, ** p<0.01, * p<0.05 compared to OVA controls.

IL-17 can induce neutrophilic inflammation by enhancing the expression of the chemotactic factor IL-8. Therefore, the mRNA expression and protein levels of KC and MIP2, the mouse orthologs of IL-8, in lung tissue were also investigated. KC and MIP2 mRNA and protein were elevated 1 day after infection and OVA sensitization in the lungs of infected, allergic, compared to uninfected, allergic groups, (Figure 8A–B). There were no differences in mRNA expression between groups at later time points. Therefore, the early induction of neutrophil influx into the lung during the development of infection-induced neutrophilic AAD is associated with early increases in neutrophil chemokine responses during infection.

### Infection induces Th17 cell differentiation and IL-17 production from Th17 cells

To investigate the mechanisms that underpin infection-induced neutrophilic AAD, we assessed the potential cellular sources of IL-17 and the role of adaptive and innate immune cells in its release.

**Figure 9:**
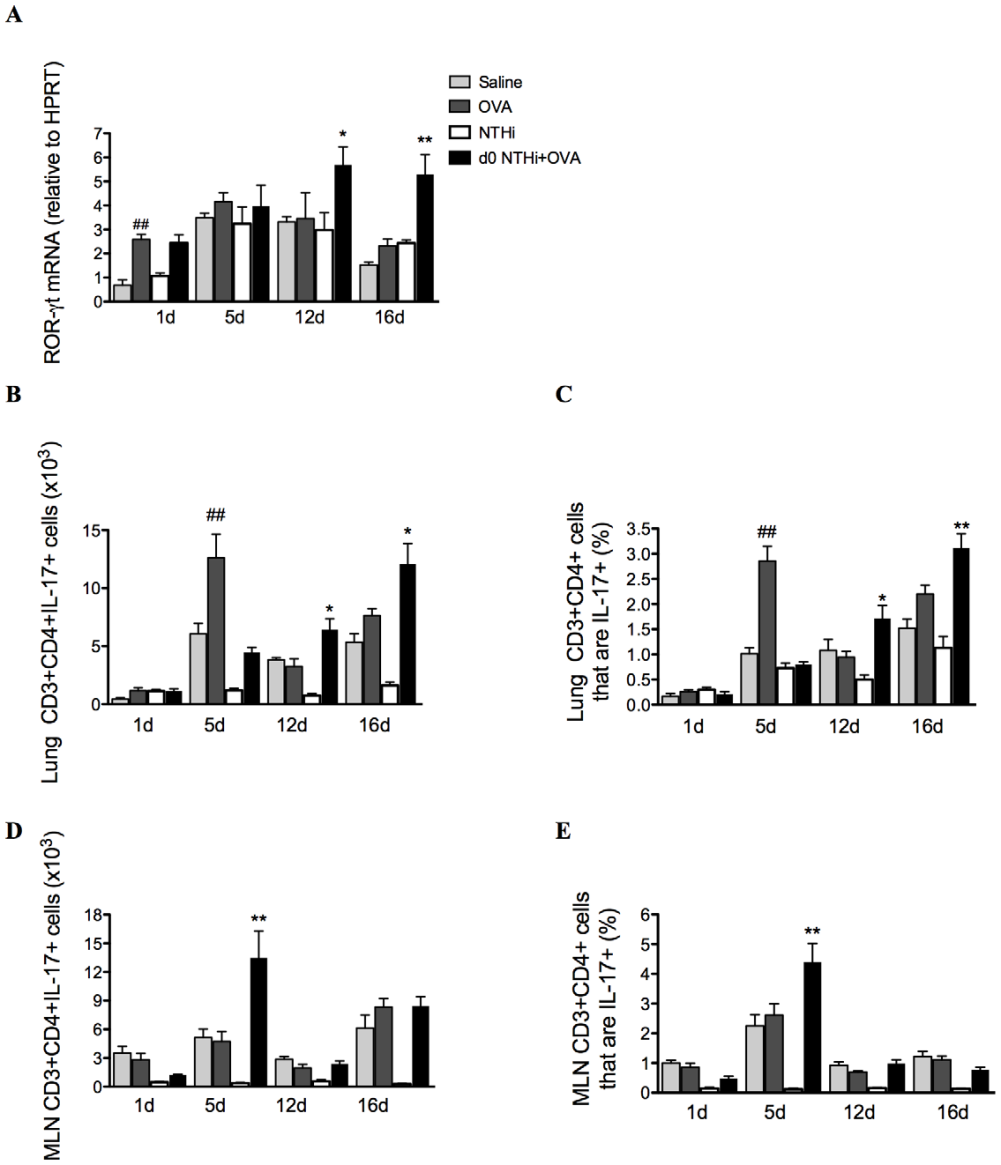
NTHi infection induces Th17 cell differentiation and IL-17 production from Th17 cells in neutrophilic AAD. Th17 cell differentiation in lung tissues was examined by determination of the expression of the transcription factor ROR-γt (A). Th17 cell numbers and proportions were quantified in lungs (B–C) and lymph nodes (D–E). Data for the corresponding NTHi control groups were obtained at the same time point after infection as data from the NTHi+OVA groups. ## p<0.01 compared to Saline controls, ** p<0.01, * p<0.05 compared to OVA controls.

The development of AAD resulted in modest increases in IL-17 factors and responses (Figure 9A–E). ROR-γt, the Th17 differentiation factor was assessed, and expression was significantly elevated after 12 and 16 days in the lungs of infected, allergic, compared to uninfected, allergic groups (Figure 9A), suggesting that there was enhanced Th17 polarization. The numbers and proportions of T cells that were Th17 cells in lung tissue and MLNs were then determined by flow cytometry. CD3^+^CD4^+^IL-17^+^ (Th17) cells were significantly increased in the lungs after 12 and 16 days in infected, allergic groups (Figure 9B–C), but in the MLNs were increased only at day 5 (Figure 9D–E). These results indicate that Th17 cells in the lungs and MLNs may be the potential adaptive immune source of IL-17 after day 5.

### Infection induces IL-17 production from macrophages and neutrophils

**Figure 10:**
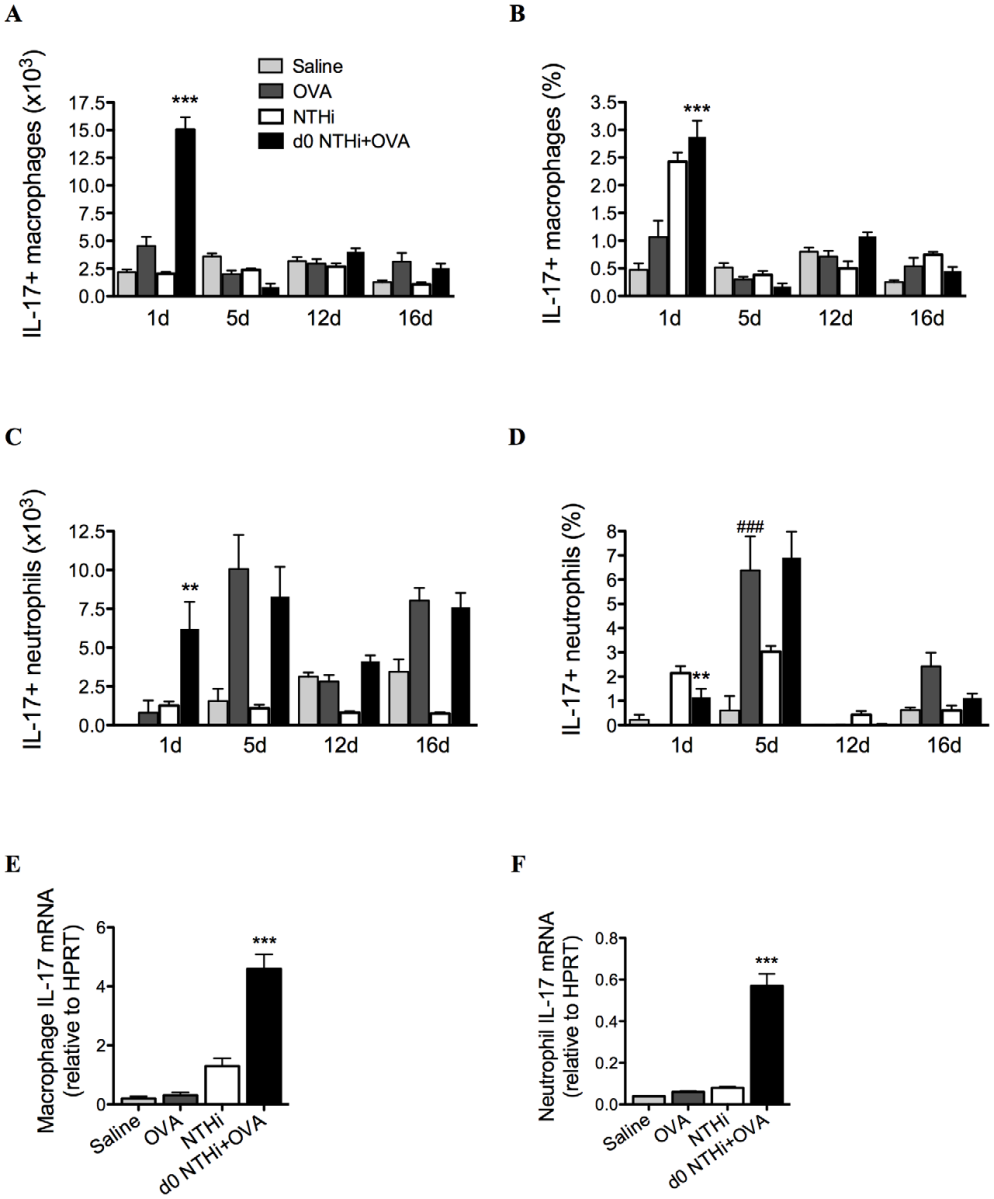
NTHi infection induces early IL-17 production from lung macrophages and neutrophils in neutrophilic AAD. To determine early cellular sources of IL-17, IL-17 producing macrophage (A–B) and neutrophil (C–D) numbers and proportions in lungs were assessed. IL-17 mRNA was also assessed in isolated lung macrophages (E) and neutrophils (F). Data for the corresponding NTHi control groups were obtained at the same time point after infection as data from the NTHi+OVA groups. ### p<0.001 compared to Saline controls, *** p<0.001, ** p<0.01 compared to OVA controls.

We then assessed which cells were the early innate sources of IL-17 on day 1. Increased numbers and proportions of pulmonary macrophages (Figure 10A–B) and to a lesser extent neutrophils (Figure 10C–D) produced increased amounts of IL-17 at early but not other time-points in infected, allergic groups compared to uninfected, allergic controls. Lung macrophages and neutrophils isolated on day 1 also had increased levels of IL-17 mRNA transcripts (Figure 10E–F).

Taken together these results demonstrate that infection induces early IL-17 responses from lung macrophages and neutrophils and later responses from Th17 cells in lungs and MLNs that are associated with neutrophil influx into the airways.

### NTHi-induced neutrophilic AAD is dependent on IL-17

**Figure 11:**
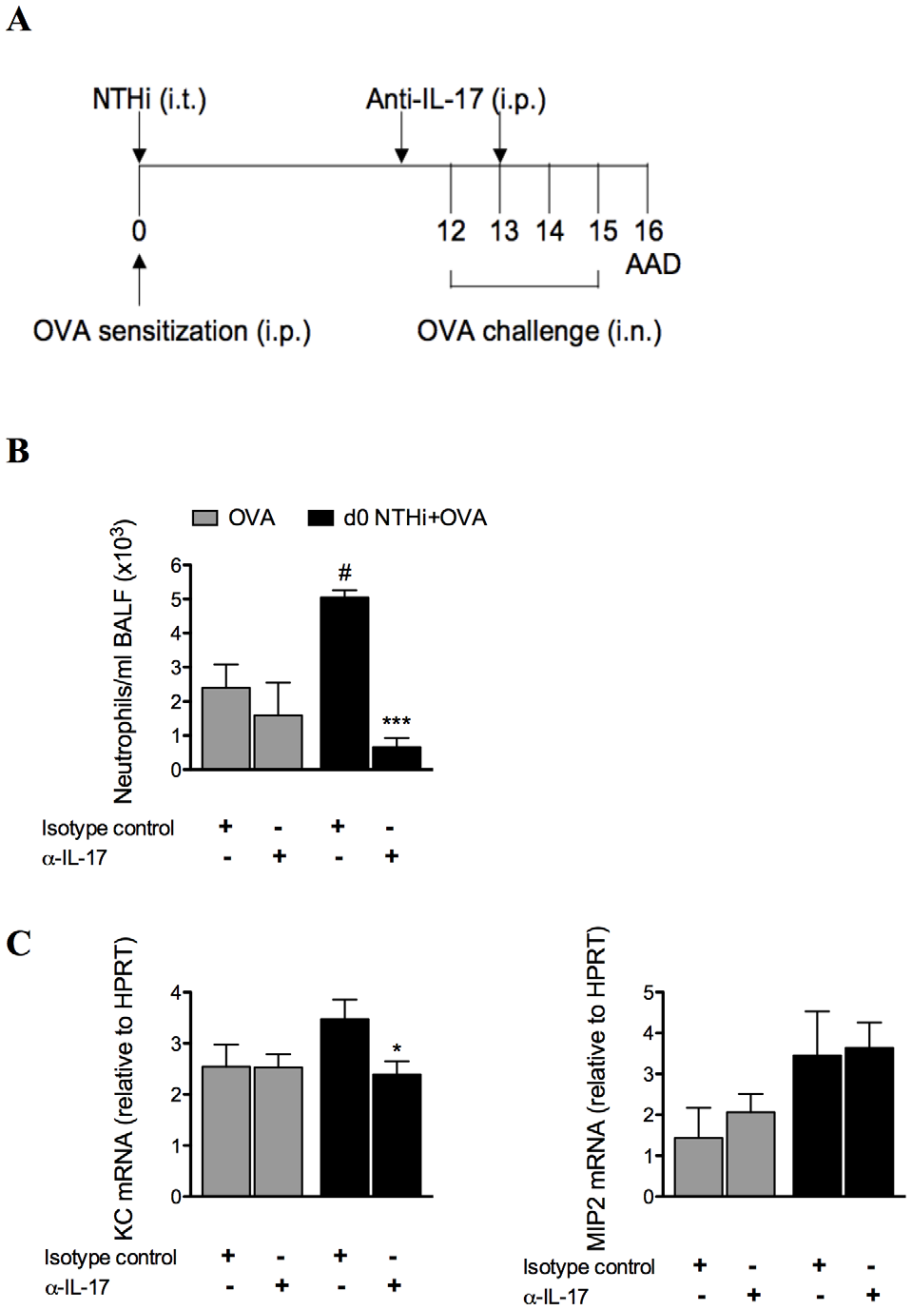
NTHi infection-induced IL-17 is required for the induction of neutrophilic AAD and is partially responsible for the effects of infection on T cell responses. To confirm the role of IL-17, anti-IL-17 monoclonal antibody was administered i.p. on days 11 and 13 of infection-induced neutrophilic AAD, and AAD was analyzed (on day 16) (A). The effects on BALF neutrophil influx (B), and KC and MIP2 mRNA expression in lung tissue (C) were assessed. # p<0.05 compared to OVA controls, *** p<0.001, * p<0.05 compared to isotype controls.

We have shown that neutrophilic inflammation in infection-induced neutrophilic AAD correlates with increased expression of IL-17 during OVA challenge. To determine whether infection-induced neutrophilic inflammation is mediated by IL-17, IL-17 was depleted in infected, allergic groups during AAD, by administration of anti-IL-17 monoclonal antibody during OVA challenge on days 11 and 13 (Figure 11A), and AAD assessed (on day 16). This approach has previously been shown to deplete IL-17 *in vivo* [21]. Importantly, IL-17 depletion significantly reduced the numbers of neutrophils in the BALF compared to isotype treatment of infected, allergic groups (Figure 11B). Significantly, neutrophil numbers were not different to those observed in uninfected, allergic groups, which were unaffected by treatment. IL-17 depletion also significantly reduced KC mRNA expression levels in the lung, but had no effect on MIP2 mRNA (Figure 11C). Anti-IL-17 treatment of infected, allergic groups also partially restored IL-5 (3.198±0.679 ng/ml in anti-IL-17 compared to 1.576±0.238 ng/ml in isotype-treated infected allergic groups p<0.01) and IL-13 (18.240±1.533 ng/ml in anti-IL-17 compared to 11.988±0.938 ng/ml in isotype-treated infected allergic groups p<0.01) responses, but had no affect on IFN-γ release. These results demonstrate that infection-induced IL-17 release is responsible for neutrophil influx into the airways and the induction of neutrophilic AAD.

## Discussion

In this study we have demonstrated for the first time that *H. influenzae* respiratory infection drives IL-17-mediated development of neutrophilic AAD. NTHi infection suppressed pulmonary and systemic eosinophilic inflammation and reduced Th2 cytokine responses and AHR in AAD. However, infection induced neutrophilic inflammation during AAD, by promoting early (innate) and late (adaptive) IL-17 responses from pulmonary macrophages and Th17 cells, respectively. This indicates that *H. influenzae* infection may modulate immune responses in asthmatics that promote the development of neutrophilic asthma.

NTHi is commonly isolated from the nasopharynx of healthy individuals, but is also associated with chronic airway diseases such as bronchiectasis [37], COPD [38], and chronic bronchitis [39]. NTHi is the bacterium most commonly isolated during COPD exacerbations, and NTHi strains isolated during these exacerbations induce higher levels of IL-8, and subsequent neutrophil recruitment to the airways, than colonizing strains [40]. Simpson and colleagues have recently demonstrated that a large proportion of neutrophilic asthmatics are colonized with *H. influenzae*, have increased innate immune activation, and 6-8 fold higher endotoxin levels compared to other asthma subtypes and healthy controls [12]. A more recent study has demonstrated that 41% of neutrophilic asthmatics assessed had a significant load of potentially pathogenic bacteria, and *H. influenzae* was identified in 60% of patients that tested positive for these bacteria [33]. We have extended these studies to show that *H. influenzae* may promote neutrophilic asthma by suppressing Th2-mediated responses that are associated with alterations in antigen-presenting cells, and by inducing potent neutrophilic inflammation that is driven by Th17 responses.

We show that infection during and after sensitization inhibits characteristic features of eosinophilic asthma. Irrespective of the time of inoculation, infection significantly reduced both local and systemic allergen-induced cytokine release from MLNs and splenocytes, as well as airway and blood eosinophil recruitment. All of these effects may lead to the suppression of AHR. Tregs are an important cell involved in immune tolerance and the suppression of inflammation [41]. We show that Treg numbers were not changed by infection, and TGF-β and IL-10 expression in the lung, which are involved in the suppression of inflammation by Tregs, were reduced by infection in AAD. These results suggest that Tregs are not involved in the suppression of cytokines or cellular inflammation. The role of infection on DC function was investigated as DCs play an integral role in the uptake and presentation of antigen to naïve T cells, and as a result direct immune responses [42]. Infection significantly decreased markers associated with antigen presentation and co-stimulation of DCs. Therefore, infection is able to alter the phenotype of antigen-presenting cells, which may affect the interaction between APCs and T cells, and result in attenuated adaptive responses to allergen.

By contrast, NTHi infection induced potent neutrophilic inflammation in the airways. Persistent airway neutrophilia is also a feature common to chronic airway diseases, such as COPD [38], chronic bronchitis [39] and bronchiectasis [43], where recurrent infection is known to play an important role in pathogenesis. Neutrophilic inflammation is often associated with acute asthma exacerbations, and in particular infection-mediated exacerbations. Indeed, several studies have shown increased neutrophilic inflammation in both viral and bacterial infection-induced exacerbations [44], [45], [46]. Here we show that NTHi induces strong neutrophilic inflammatory responses, and may be involved in the development of neutrophilic asthma through the induction of neutrophilic inflammation. We demonstrate that immune responses that lead to the development of NTHi-induced neutrophilic AAD occur in two phases. The first involves innate immune activation during infection that is likely to result in neutrophil chemoattraction to the airways. NTHi infection during OVA sensitization resulted in a significant neutrophil influx to the airways 1 day after infection, a two-fold increase compared to NTHi infection alone. This correlated with the early production of KC, MIP2 and IL-17 in the lungs. These neutrophils and to a greater extent macrophages were able to produce significantly more IL-17 than those from infected, nonallergic and uninfected allergic controls. Therefore these cells, particularly macrophages, may be sources of early (innate) IL-17 release. This observation may have important implications for other diseases where the innate source of IL-17 has not yet been identified.

The second phase involves adaptive immune responses during allergen challenge resulting in increased infection-mediated Th17 responses. During the challenge phase, days 12-15, there was a significant upregulation of ROR-γt and IL-17 mRNA in the lungs of infected allergic groups compared to infected, nonallergic and uninfected allergic controls. These results directly correlated with increases in Th17 cells in the lungs of infected, allergic groups. The increased production of IL-17 from T cells in conjunction with increases in ROR-γt expression suggest that infection drives Th17 responses that preferentially induce IL-17 production and neutrophilic inflammation during subsequent allergen challenge.

Collectively, our data suggest that infection induces early responses, involving neutrophils, KC, MIP2 and IL-17 expression that may prime the host for enhanced Th17-mediated neutrophilic responses upon later allergen challenge, which subsequently induces neutrophilic AAD. Our findings are consistent with data from a recent study by Bullens *et al*., which showed that increased IL-17 responses in asthmatics correlate with increased neutrophil numbers in sputum [15]. Hellings *et al.*, [21] demonstrated that IL-17 is important in lung neutrophil recruitment in response to an allergen, while Ye *et al*., [29] showed that IL-17 responses and signalling through the IL-17R is vital for neutrophil recruitment and host defence against *Klebsiella pneumoniae* infection. Here we extend these findings by demonstrating that *H. influenzae* infection-induced IL-17 responses in AAD may play a role in driving neutrophilic inflammation in asthma. We recently demonstrated that chlamydial respiratory infection is also able to drive neutrophilic asthma [47]. Chlamydial infection suppressed Th2-mediated eosinophilic inflammation and promoted neutrophilic inflammation and AAD. Neutrophilic asthmatics are resistant to corticosteroid treatment, which is the mainstay of asthma therapy [10], and with evidence that asthmatics with infection are more resistant to steroids than asthmatics with no infection [48], alternative therapies are needed for infection-induced neutrophilic asthma.

It is possible that either infection alone or the synergistic effects of infection and AAD are required for the induction of the neutrophilic AAD phenotype. NTHi infection alone did increase neutrophil influx into the airways (p<0.01), IL-17 mRNA expression in the lung (p<0.05) and the percentages of lung macrophages and neutrophils producing IL-17 (both p<0.001) 1 day after infection, compared to uninfected, nonallergic (Saline) controls. These effects would be expected as they are normal responses to infection; however, they may contribute to the establishment of a pro-neutrophilic environment and the subsequent development of neutrophilic AAD upon allergen challenge. However, NTHi infection does not persist past 10 days, and yet is able to modify AAD after 16 days. It is, therefore, likely that the major affect of the infection is to induce persistent immune changes, that continue even after the clearance of the infection, that synergize with allergen exposure to drive neutrophilic AAD. This mechanism has recently been proposed in humans [49].

OVA sensitization at the time of, or prior to, infection, and subsequent OVA challenge does not affect NTHi load. When infection occurs during sensitization, bacteria are still cleared by day 16, and; when infection occurs 10 days after sensitization, bacterial recovery at the end of the protocol (i.e. 5 days later), is the same as that in the infected, non-allergic group (data not shown).

It is likely that the induction of a neutrophilic phenotype is specific to a subset of infectious agents, particularly NTHi and *Chlamydia* [12], [33], [50]. In other studies we have investigated the impact on AAD of an unrelated respiratory pathogen and commensal bacterium, *Streptococcus pneumoniae*, which is not associated with neutrophilic asthma. We show that *S. pneumoniae* infection or components do not induce changes in IL-17 responses or increase neutrophilic inflammation with infection or component administration either before, during or after the induction of AAD [51], [52]. Both NTHi- and *Chlamydia-*induced neutrophilic AAD, may potentially be driven by conserved pathogen-associated molecular patterns (PAMPs), such as lipopolysaccharide (LPS) or CpGs. Numerous studies have investigated the effects of LPS and CPG in AAD, and the interactions are complex. LPS is highly variable and different types and levels of LPS have different effects in AAD. Low dose LPS administration during sensitization promotes Th2 responses and is a risk factor for severe asthma [53], [54], while high doses decrease Th2 responses and induce non-eosinophilic inflammation [55], [56], [57], [58]. *Chlamydia*-derived LPS is atypical and is 1,000 fold less immunogenic compared to other bacterial-derived LPS [59], and therefore, is unlikely to be the cause of *Chlamydia*-induced neutrophilic AAD. CpGs when given together with antigen in established disease induce Th1 and regulatory T cell responses that suppress the features of AAD including Th2 responses and AHR [60], [61], [62]. We have shown that depleting neutrophils during a *Chlamydia* infection inhibits the development of neutrophilic AAD, by contrast IL-17 responses drive NTHi-induced neutrophilic AAD. Therefore, we suggest that it is specific immune responses to these, as well as potentially other infections, which are driven by as yet unidentified factors in the infections, that are the mechanisms that drive neutrophilic AAD [47]. Our studies do not rule out LPS, CpGs, or other PAMPs as drivers of the neutrophilic phenotype and further research is required to elucidate this possibility.

Importantly we have shown that infection-induced neutrophilic AAD and the suppression of Th2 responses are dependent upon IL-17. Depletion of IL-17 with anti-IL-17 monoclonal antibody during AAD prevented the development of infection-induced neutrophilic AAD. This suggests that IL-17 is critical in the recruitment of neutrophils and may suppress Th2 responses in infection-induced neutrophilic inflammation and neutrophilic asthma. Wakashin *et al*., demonstrated that adoptive transfer of antigen-specific Th17 cells induced airway neutrophil recruitment, which supports our data [63]. Little is known about how Th17 and Th2 cells interact with or regulate each other. We demonstrate that infection inhibits cytokine release compared to uninfected allergic controls, and that anti-IL-17 treatment partially restored these effects, while having no effect on eosinophil recruitment (data not shown). This suggests that other mechanisms are also involved in the suppression of Th2 responses by NTHi infection, which requires further investigation. Several recent studies have investigated the relationship between Th17 and Th2 cells. Schnyder-Candrian *et al*., showed that the administration of rIL-17 in a murine model of AAD significantly reduced allergen-induced eotaxin, thymus and activation-regulated chemokine (TARC) and IL-5, thereby reducing eosinophilic inflammation [64], while another study showed that inhibiting IL-17 in AAD also reduced airway eosinophils, neutrophils, AHR, and Th2 cytokines [65]. These data suggest that IL-17 may suppress or promote eosinophilic inflammation, but the mechanisms that drive these different effects remain unknown. Our data is in agreement with Schnyder-Candrian *et al*., who suggest that IL-17 interferes with DC activation and antigen uptake, which prevents T cell activation and reduced IL-4, -5 and -13 production, leading to suppressed allergic responses. Interestingly, a recent study has shown a CD4^+^ T cell subtype that expresses both Th17 and Th2 cytokines, including IL-4, IL-5, IL-13, IL-17 and IL-22, and this subset is increased in asthmatics compared to healthy controls [66]. However, these cells have thus far only been found in the periphery, and confirmation of their presence is needed in BAL, sputum and/or bronchial biopsies.

In conclusion, we show that *H. influenzae* infection may be involved in the development of neutrophilic asthma. Infection suppressed features of Th2-mediated eosinophilic AAD, while inducing features of neutrophilic asthma that are mediated by infection-induced IL-17. Therefore, infection-induced IL-17 responses may play a major role in the pathogenesis of neutrophilic asthma. Our studies indicate the important role of infection in driving neutrophilic asthma-like disease, and identify new areas of investigation that may enhance the understanding of disease progression. Developing new treatments targeting infection may lead to better management of individuals with this disease phenotype.

## Methods

### Ethics statement

This study was carried out in strict accordance with the recommendations in the NSW Animal Research Regulation 2005, and the Australian Code of Practice for the care and use of animals for scientific purposes (National Health and Medical Research Council). All protocols were approved by the Animal Care and Ethics Committee of the University of Newcastle (permit number 987/0111). All surgery was performed under sodium pentobarbital anaesthesia, and all efforts made to minimize pain and suffering.

### AAD model

Six to eight week old female BALB/c mice were used. Mice were sensitized by intraperitoneal (i.p.) injection, with OVA (50 µg, Sigma-Aldrich, Castle Hill, NSW, Australia) with the Th2-inducing adjuvant Rehydrogel (1mg, in 200 µl sterile saline, Reheis, Berkeley Heights, USA). On days 12 to 15 mice were challenged intranasally (i.n.) with OVA (10 µg, 50 µl) and AAD was assessed on day 16 [67]. Controls were sham sensitized to saline.

### NTHi infection model

NTHi (NTHi-289) glycerol stocks were plated onto chocolate agar plates (Oxoid, SA, Australia), grown overnight (37°C, 5% CO_2_), then washed off the plate and suspended in sterile PBS. To determine the effects of infection, mice were inoculated i.t. with 5x10^5^ CFU NTHi (in 30 µl PBS) during (Day 0) or after (Day 10) OVA sensitization. Controls were infected but not exposed to OVA. In preliminary studies we determined that this inoculum induced an infection from which the mice recovered and could be used to study the effects of infection on AAD.

### Cellular inflammation

BALF was collected and processed as previously described [68]. Briefly, the left lung was tied off and the right lung was washed twice with Hank's buffered salt solution (700 µl, HBSS; Trace Scientific, Noble Park, Vic, Australia). Cells were pelleted and resuspended in red blood cell lysis buffer, washed and resuspended in HBSS, then cytocentrifuged (300*g*, 5 min, ThermoFisher Scientific, Scoresby, Vic, Australia) onto microscope slides. Blood smears were prepared from a drop of whole blood. BALF and blood cells were stained with May-Grunwald-Giemsa, and differential leukocyte counts were enumerated using light microscopy [68].

### Bacterial recovery

Right lobes of lungs, from which BALF had been obtained, were aseptically removed and homogenized in 1ml of sterile PBS. Serial dilutions of BALF and lung homogenates were prepared in sterile PBS, plated onto chocolate agar plates and incubated overnight (37°C, 5% CO_2_). Colonies were enumerated and bacterial numbers per right lung calculated.

### Lung function

AHR was measured in response to increasing doses of aerosolized methacholine, by whole body invasive plethysmography as previously described [47]. Briefly, mice were anaesthetized and tracheas were cannulated and attached to a ventilator. Peak dynamic compliance and transpulmonary resistance were assessed by analysis of pressure and flow waveforms following challenge with increasing doses of aerosolized methacholine (Sigma-Aldrich).

### T cell cytokines

Supernatants from lung draining MLN T cells were restimulated with OVA (200 µg/ml) or ethanol-killed NTHi (2×10^7^ CFU/ml) and cultured for six days (5% CO_2_, 37°C, 1x10^6^ cells per well). After culture supernatants containing soluble factors were recovered and analyzed for IL-5, IFN-γ, (BD Biosciences, North Ride, NSW, Australia), IL-13, IL-17A and IL-22 (R&D Systems, Minneapolis, MN, USA) by ELISA, according to manufacturer's instructions [69].

### Lung protein cytokines

Whole lungs were homogenized in RIPA buffer (1ml, Sigma-Aldrich) and incubated on ice for 5 mins. Cells were pelleted and the supernatant recovered and analyzed for KC and MIP2 by ELISA, (R&D Systems), according to manufacturer's instructions.

### Cytokine expression in lungs

RNA was TRIZOL extracted from whole lung homogenates according to manufacturer's instructions (Invitrogen, Mount Waverly, Vic, Australia). Target gene expression was determined relative to the reference gene hypoxanthine-guanine phosoribosyltransferase (HPRT) [69]. Primers used were IL-17, Fwd 5′-aaacatgagtccagggagagcttt-3′, Rev 5′-actgagcttcccagatcacagagg-3′; ROR-γt, Fwd 5′- ccgctgagagggcttcac-3′, Rev 5′- tgcaggagtaggccacattaca-3′; MIP2, Fwd 5′- ctagctgcctgcctcattctac-3′, Rev 5′- caacagtgtacyyacgcagacg-3′; KC Fwd 5′- cttggggacaccttttagca-3′, Rev 5′- gctgggattcacctcaagaa-3′; TGF-β Fwd 5′- cccgaagcggactactatgctaaa-3′, Rev 5′- ggtaacgccaggaattgttgctat-3′; IL-10, Fwd 5′-catttgaattccctgggtgagaag-3′, Rev 5′- gccttgtagacaccttggtcttgg-3′; and HPRT Fwd 5′- aggccagactttgttggatttgaa-3′, Rev 5′- caacttgcgctcatcttaggcttt-3′.

### Flow cytometry

Single cell suspensions of MLNs and collagenase-D digested lungs were prepared. IL-17 producing cells were identified by stimulation with phorbol 12-myristate 13-acetate (PMA, 0.1 µg/ml) and ionomycin (1 µg/ml, Sigma-Aldrich) in the presence of Brefeldin A (8 µg/ml, Sigma-Aldrich) for 4 hours [70]. Cells were incubated with Fc block for 15mins, then stained for surface markers CD4, CD3, CD11b, Gr-1 (BD Bioscience), or F4/80 (eBioscience, San Diego, CA, USA), fixed with 4% paraformaldehyde (PFA), permeabilized with 0.1% saponin, and stained for intracellular IL-17 (or isotype control rat IgG2a, eBioscience). Tregs were identified using surface markers CD4, CD25 and a staining kit for intracellular FoxP3 (or IgG_2a_ isotype control) according to manufacturer's instructions (eBioscience). pDCs were characterized as CD11c^low^CD11b^-^B220^+^, and mDCs characterized as CD11c^+^CD11b^+^B220^−^ (BD Bioscience); using MHCII and CD86 (R&D Systems) for activation and co-stimulation status. All cells were analyzed using a FACS Canto (BD Bioscience) [47].

### Lung macrophage and neutrophil separation and mRNA isolation

Single cell suspensions of collagenase-D digested lung tissue were prepared, and resuspended in red blood cell lysis buffer. Resuspended cells were either incubated overnight (5% CO_2_, 37°C, 1x10^6^ cells per well) and macrophages isolated by adherence to culture plates; or were put through a mouse neutrophil enrichment kit (Stemcell Technologies, Melbourne, Vic, Australia) and neutrophils isolated by negative selection following manufacturer's instructions. mRNA was purified from these macrophages and neutrophils using a PureLink RNA mini kit (Invitrogen) according to manufacturer's instructions.

### Depletion of IL-17 during infection-induced neutrophilic AAD

Monoclonal anti-IL-17A neutralizing antibody (clone 50104, rat IgG_2a_) was administered by i.p. injection (100 µg/mouse, eBioscience) on days 11 and 13, and features of AAD were assessed on day 16. Control groups were uninfected and treated with anti-IL-17 or treated with IgG_2a_ isotype control antibody [47].

### Statistics

Results are presented as mean ± standard error of the mean (SEM) from 6–8 mice, in duplicate. Significance was determined by one-way ANOVA or Student *t*-test (GraphPad Software, CA, USA).
